# One-year delayed effect of fog on malaria transmission: a time-series analysis in the rain forest area of Mengla County, south-west China

**DOI:** 10.1186/1475-2875-7-110

**Published:** 2008-06-19

**Authors:** Linwei Tian, Yan Bi, Suzanne C Ho, Wenjie Liu, Song Liang, William B Goggins, Emily YY Chan, Shuisen Zhou, Joseph JY Sung

**Affiliations:** 1 Stanley Ho Center for Emerging Infectious Diseases, School of Public Health, Chinese University of Hong Kong, Hong Kong, PR China; 2 Yunnan Province Center for Disease Control and Prevention, Kunming, PR China; 3 Department of Community and Family Medicine, School of Public Health, Chinese University of Hong Kong, Hong Kong, PR China; 4 Xishuangbanna Tropical Botanical Garden, Chinese Academy of Sciences, Kunming, PR China; 5 College of Public Health, Ohio State University, Columbus, Ohio, USA; 6 Division of Biostatistics, School of Public Health, Chinese University of Hong Kong, Hong Kong, PR China; 7 National Malaria Office, National Institute for Parasitic Diseases, Shanghai, PR China

## Abstract

**Background:**

Malaria is a major public health burden in the tropics with the potential to significantly increase in response to climate change. Analyses of data from the recent past can elucidate how short-term variations in weather factors affect malaria transmission. This study explored the impact of climate variability on the transmission of malaria in the tropical rain forest area of Mengla County, south-west China.

**Methods:**

Ecological time-series analysis was performed on data collected between 1971 and 1999. Auto-regressive integrated moving average (ARIMA) models were used to evaluate the relationship between weather factors and malaria incidence.

**Results:**

At the time scale of months, the predictors for malaria incidence included: minimum temperature, maximum temperature, and fog day frequency. The effect of minimum temperature on malaria incidence was greater in the cool months than in the hot months. The fog day frequency in October had a positive effect on malaria incidence in May of the following year. At the time scale of years, the annual fog day frequency was the only weather predictor of the annual incidence of malaria.

**Conclusion:**

Fog day frequency was for the first time found to be a predictor of malaria incidence in a rain forest area. The one-year delayed effect of fog on malaria transmission may involve providing water input and maintaining aquatic breeding sites for mosquitoes in vulnerable times when there is little rainfall in the 6-month dry seasons. These findings should be considered in the prediction of future patterns of malaria for similar tropical rain forest areas worldwide.

## Background

Malaria is a major public health burden in the tropics [1] with the potential to significantly increase in response to climate change [2]. Analyses of data from the recent past can elucidate how short-term variations in weather factors affect malaria transmission. These findings can be applied in a modeling exercise to estimate future patterns of malaria. Over the past century the world has warmed by 0.6°C [3], with a range of ecological consequences [4]. The possible linkage between global warming and the increase in malaria incidence or its geographic spread has been extensively debated [5-7]. The current evidence is insufficient to clearly attribute the increase of malaria incidence or its geographic spread in the east African highlands to local warming [8]. The relationship between climate and malaria may be highly dependent upon local scale parameters, and it is not always possible to extrapolate the relationship to a broader spatial scale. Moreover, caution is needed when the empirical evidence of short-term climate variation and malaria transmission is applied to the estimation of future impacts of climate change. Investigations that examine the consistency of climate and malaria relationships in different societal and regional contexts can improve our understanding of the linkages between climate and malaria transmission and provide a stronger scientific foundation for predicting future patterns of malaria [9].

**Figure 1:**
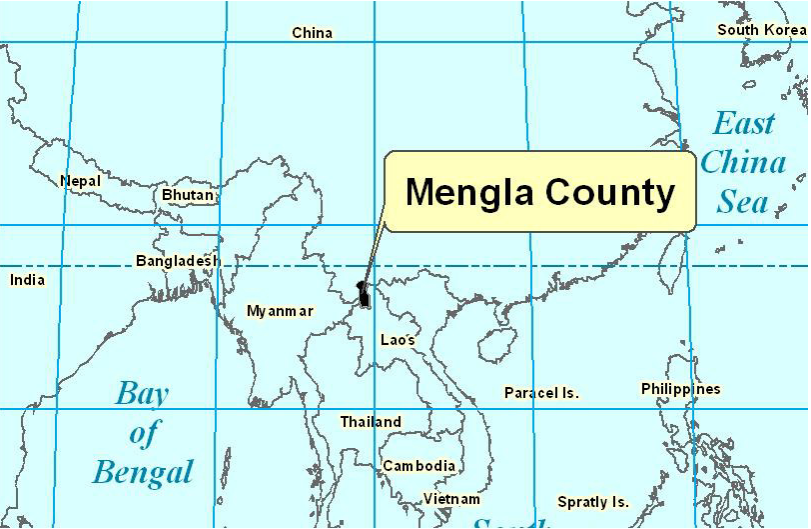
Location of Mengla County, China.

Although the linkage between climate variability and malaria transmission has been widely studied in the east African highlands and other areas [6,10-14], few studies in this regard have been conducted in the tropical areas of southern China and south-east Asia. In this study, the potential impact of climate variability on the transmission of malaria in a tropical county of China was examined. Malaria is still a major public health issue in China, especially in Yunnan and Hainan provinces, despite nationwide malaria control efforts in the past decades [15]. In 2005, malaria incidence was 49.5/100,000 in Yunnan Province, where a total of 15,072 cases and 38 deaths were reported. The ratio of *Plasmodium vivax *malaria cases to *Plasmodium falciparum *malaria cases was 4:1. Mengla County (21°09'-22°24'N, 101°05'-101°50'E) of Yunnan Province is situated just south of the tropic of Cancer, bordering Laos on the east, south, and south-west, and Myanmar on the west (Figure 1). It has an area of 7,093 km^2^, is mostly mountainous, and has a population of 0.2 million. Its elevation ranges from 480 m to 2,023 m. Mengla County has one of the highest malaria incidence rates in China; during 1994–1998, its annual malaria incidence rate, 400.4/100,000, was the sixth among the 2,353 counties of China [16].

The purpose of the current study was to examine the effects of weather factors on the transmission of malaria in Mengla County by using auto-regressive integrated moving average (ARIMA) models. Ecological time-series analysis has been used extensively to study the effect of climate variability on infectious diseases [12,17,18]. ARIMA models are useful tools to analyze time-series data containing ordinary or seasonal trends [19]. The current analysis was based on malaria incidence and weather factor data from Mengla County for the 1971–1999 period. The weather factors included minimum temperature, maximum temperature, rainfall, humidity, and fog. The monthly or annual fog day frequency was used as an index variable for fog abundance.

## Methods

Malaria incidence data were obtained from Yunnan Province's Center for Disease Control and Prevention. As a national malaria surveillance location, Mengla County has kept complete malaria records for nearly four decades. *Plasmodium vivax *malaria is predominant in this county, but *P. falciparum *infections also exist. Overall malaria incidence was used in this study. All residents in the county during the 1971–1999 period were treated as the study population. Weather data including monthly rainfall, minimum temperature, maximum temperature, relative humidity, and fog day frequency were retrieved from the Yunnan Bureau of Meteorology. A fog day is defined as a day when visibility is 1,000 m or less for more than 15 min. Before conducting the time-series analysis, logarithmic transformation was applied to the malaria incidence time series to assure the normality and homogeneity of variance of the residuals.

ARIMA models were used to evaluate the relationship between weather factors and monthly malaria incidence. An ARIMA model was fit first to the predictor variable. The model was then applied to the dependent variable before the two series were cross-correlated to determine whether an association exists. Modeling with ARIMA involves the estimation of a series of parameters to account for the inherent dynamics in the time series, including the trends and autoregressive and moving average processes. The general model introduced by Box and Jenkins [19] includes autoregressive and moving average parameters, and explicitly includes differencing in the formulation of the model. An ARIMA (*p, d, q*) model comprises three types of parameters: the autoregressive parameters (*p*), number of differencing passes (*d*), and moving average parameters (*q*). The multiplicative seasonal ARIMA (*p, d, q*)(*P, D, Q*)_*s *_model is an extension of the ARIMA method to time series in which a pattern repeats seasonally over time. Analogous to the simple ARIMA parameters, the seasonal parameters are: seasonal autoregressive (*P*), seasonal differencing (*D*), and seasonal moving average parameters (*Q*). The length of the seasonal period is represented by *s*.

Each of the weather input series, at lags of one to 12 months, respectively, was fitted into the seasonal ARIMA model of monthly malaria incidence to screen for potential weather predictors of malaria incidence. Those input series significantly associated with malaria incidence, with a p-value of less than 0.10, were singled out to fit the best multivariate ARIMA model. The Ljung-Box Q test was applied to ascertain whether the residual series were white noise. The conditional least squares method was applied in the ARIMA procedure of SAS (SAS Institute, Inc., Cary, North Carolina). The selection of ARIMA processes was conducted using Akaike's information criterion (AIC), which measures how well the model fits the series. At the time scales of years, each of the weather input series, at lags of 0 and 1 year, respectively, was fitted into the ARIMA model of annual malaria incidence.

In order to examine whether the association between weather and malaria remains constant or whether it is particularly strong in certain months, the response and predictor time series were also modeled separately and the cross-correlations between their residual series were examined subsequently [20,21]. One property of a white noise series (i.e., residual series after ARIMA modeling) is that a time series composed of sequences of a white noise series is again a white noise series [21]. This study took advantage of this property to examine in more detail the associations between weather factors and malaria incidence. Specific sequences (e.g., January of each year) of the residual time series constitute a new white noise series. Moving a one-month time frame throughout the year can result in 12 cross-correlations, which can be used to examine whether the association between weather and malaria remains constant or whether it is particularly strong in certain months. All analyses were performed using SAS for Windows, version 9, software (SAS Institute, Inc., Cary, North Carolina).

## Results

**Figure 2:**
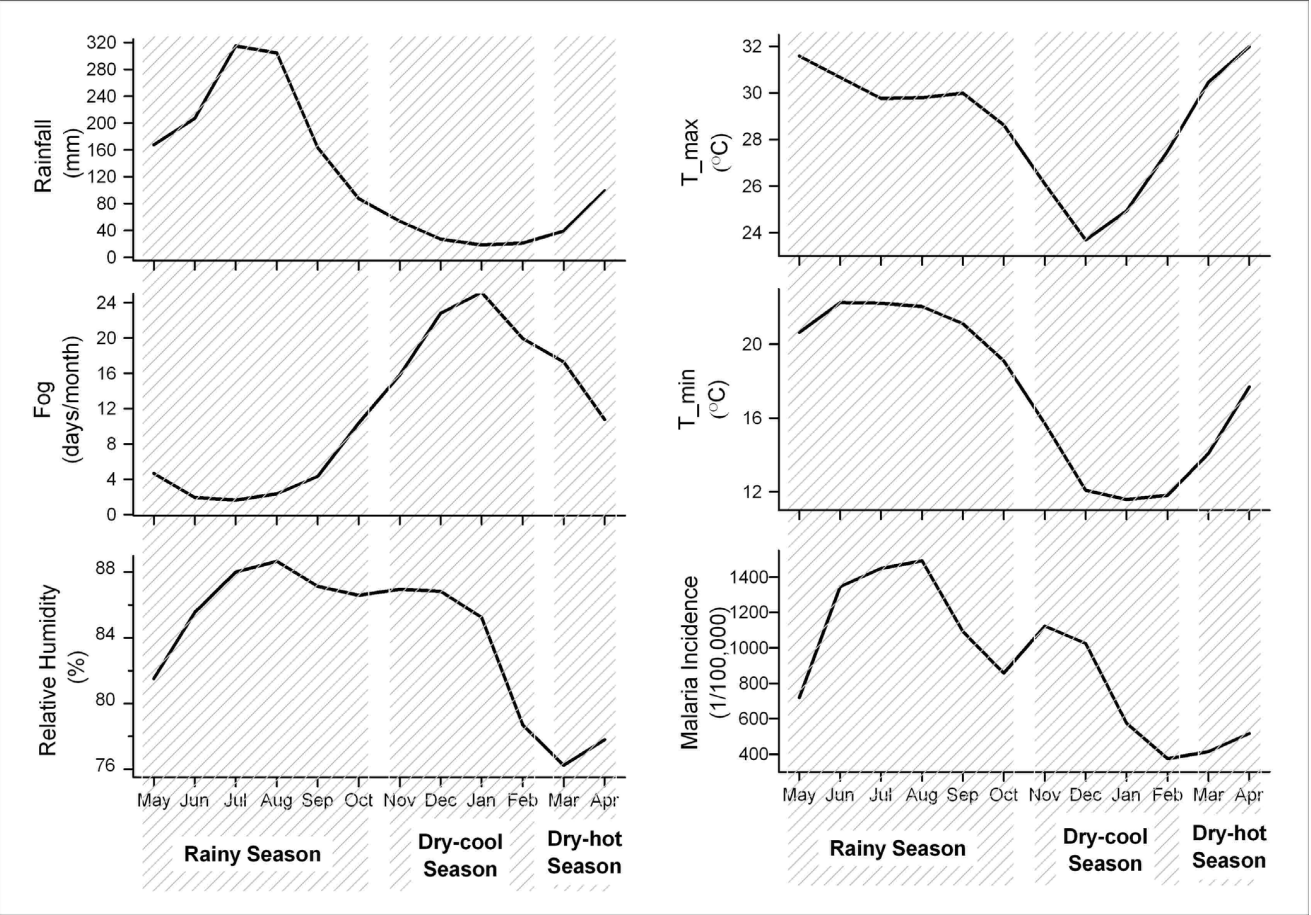
Average monthly weather measurements and malaria incidence rates (1971–1999) in Mengla County.

Figure 2 shows the average seasonal pattern of rainfall, fog day frequency, and malaria incidence in Mengla County. In this tropical rain forest area, there are three seasons: rainy season (May-October), dry-cool season (November-February), and dry-hot season (March-April). Seasonal variations are apparent in malaria incidence and the two weather variables: rainfall and fog day frequency. Rainfall is the highest in the rainy season and lowest in the dry-cool season. Fog persists in the dry-cool season and the dry-hot season, and occurs only occasionally in the rainy season. Besides the rainy season peak (in July and August) of malaria incidence, there is also a peak in November and December in the dry-cool season.

By fitting each of the weather input series, at lags of one month to 12 months, respectively, in the ARIMA model of monthly malaria incidence, a total of six input series were found to be significantly associated with malaria. Two input series, humidity and rainfall at a four-month lag, are inversely associated with malaria incidence. The four input series positively associated with malaria include: maximum temperature at a lag of four months, minimum temperatures at lags of one month and two months, and the fog day frequency at a lag of seven months. Different combinations of monthly temperatures, rainfall, humidity, and fog were added to the models as input series. Of all the models tested, the seasonal ARIMA *(1,1,1)(0,1,1)*_12 _model for malaria incidence fits the data best according to AIC and goodness-of-fit criteria.

Model II in Table 1 is the best fitting one among three models tested based on the monthly time series data. The local moving average parameter is 0.517, the seasonal moving average is 0.795, and the autoregression is -0.167, all of which are statistically significant. Malaria incidence is positively associated with maximum temperature at a lag of four months (*β *= 0.018, *p *= 0.021), minimum temperature at a lag of one month (*β *= 0.032, *p *= 0.002) and two months (*β *= 0.028, *p *= 0.006), and the fog day frequency at a lag of seven months (*β *= 0.004, *p *= 0.021). Model I shows that the inclusion of the additional covariates of humidity (*β *= -0.003, *p *= 0.531) and rainfall (*β *= -0.0001, *p *= 0.478) does not improve the model fit, and that these two variables are not significantly associated with malaria incidence. In summary, the best fitting model includes minimum temperature, maximum temperature, and fog day frequency as the predicting variables for the monthly malaria incidence.

**Table 1:** ARIMA regression of the logarithmic monthly malaria incidence (1971–1999) on the weather factors in Mengla, China

	Model I			Model II		
	
	*β*	S.E.	*p*	*β*	S.E.	*p*
Moving average	0.520	0.049	<.0001	0.517	0.049	<.0001
Seasonal moving average	0.799	0.035	<.0001	0.795	0.035	<.0001
Auto-regression	-0.165	0.057	0.004	-0.167	0.057	0.004
Humidity (lag 4)	-0.003	0.005	0.531			
Rainfall (lag 4)	-0.0001	0.0001	0.478			
T_max (lag 4)	0.012	0.010	0.212	0.018	0.008	0.021
T_min (lag 1)	0.032	0.010	0.002	0.032	0.010	0.002
T_min (lag 2)	0.027	0.010	0.008	0.028	0.010	0.006
Fog frequency (lag 7)	0.004	0.002	0.020	0.004	0.002	0.021
AIC		-202.2			-204.8	

**Figure 3:**
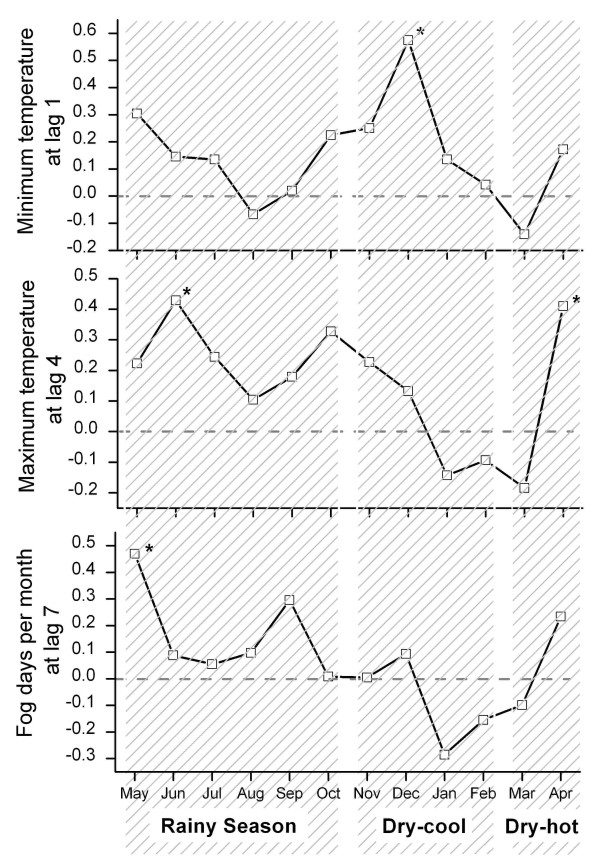
Cross-correlations between weather factors and malaria incidence based on moving time frames of one month after modeling with the autoregressive integrated moving average, in Mengla County from 1971 to 1999. (* p < 0.05)

Figure 3 shows that the strength of the association between these weather factors and malaria incidence is not constant throughout the year. The cross-correlation between malaria incidence and minimum temperature at a lag of one month is the strongest in December (*rho *= 0.575, *p *= 0.001). A higher minimum temperature in November predicts an elevated malaria incidence in December. For minimum temperature at a lag of two months, the cross-correlation is the highest in February (*rho *= 0.357, *p *= 0.067). A minimum temperature in December is positively associated with malaria incidence in February of the following year. Taken together, the effect of minimum temperature on malaria incidence appears stronger in the cool months than in the hot months.

The cross-correlation between malaria and maximum temperature at a lag of four months is particularly strong in April and June (*rho *= 0.410, *p *= 0.034; *rho *= 0.429, *p *= 0.023, respectively). The maximum temperature in the cool months is positively associated with malaria incidence after a four-month interval. In other months, the cross-correlation is not statistically significant. To generalize, the effect of maximum temperature in cool months is stronger than that in hot months.

The overall association between malaria incidence and fog at a lag of seven months is driven particularly by the cross-correlation in May (*rho *= 0.470, *p *= 0.013). The cross-correlation is not statistically significant in other months. The fog day frequency in October is positively associated with malaria incidence in May of the following year. The seven-month lead time goes beyond the six-month dry season. When rainfall reduces by the end of the rainy season, fog day frequency starts to increase. It is actually the fog day frequency in the late rainy season that affects malaria incidence in the early rainy season of the following year.

**Figure 4:**
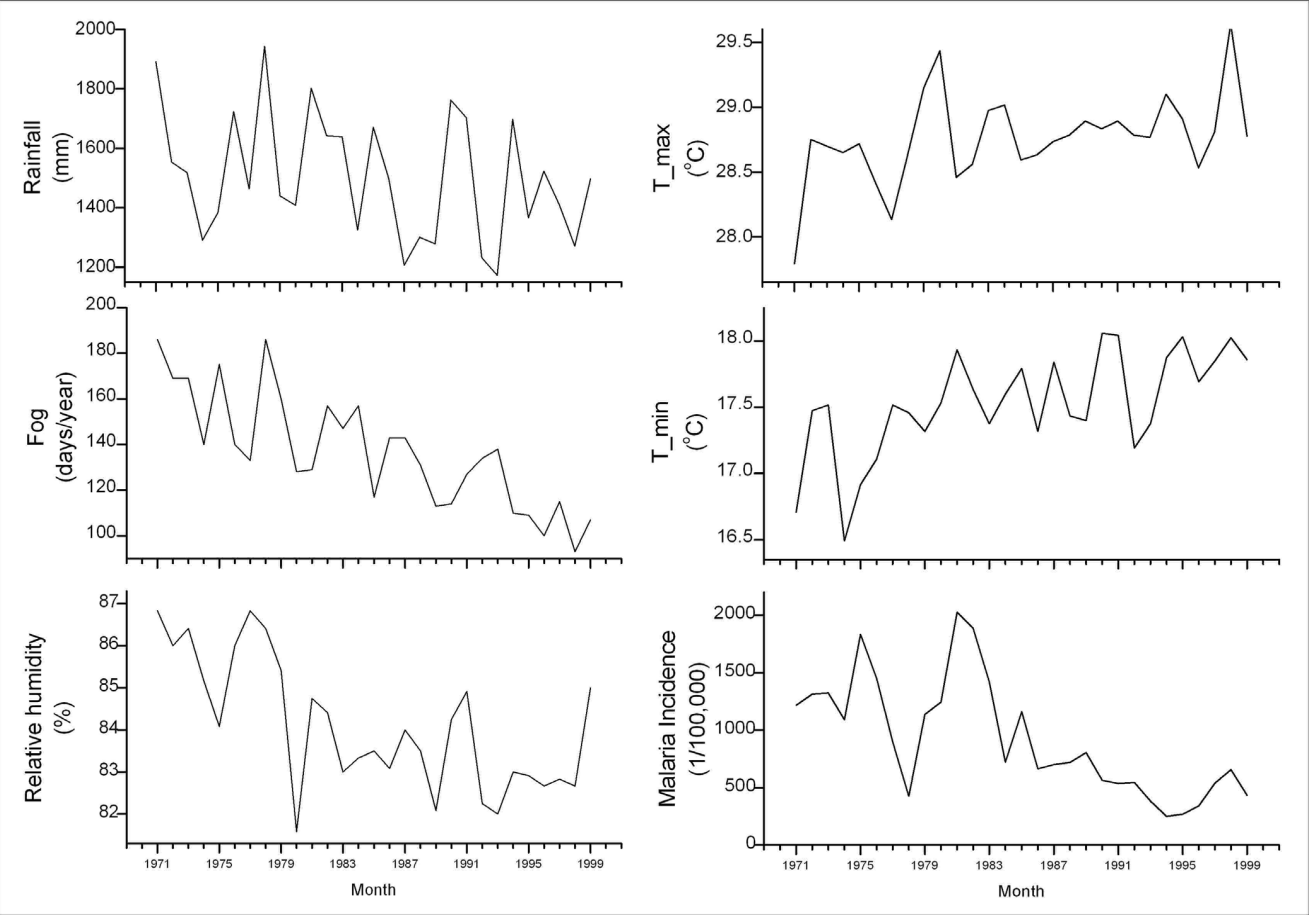
Weather factors and malaria incidence in Mengla County, 1971–1999.

**Figure 5:**
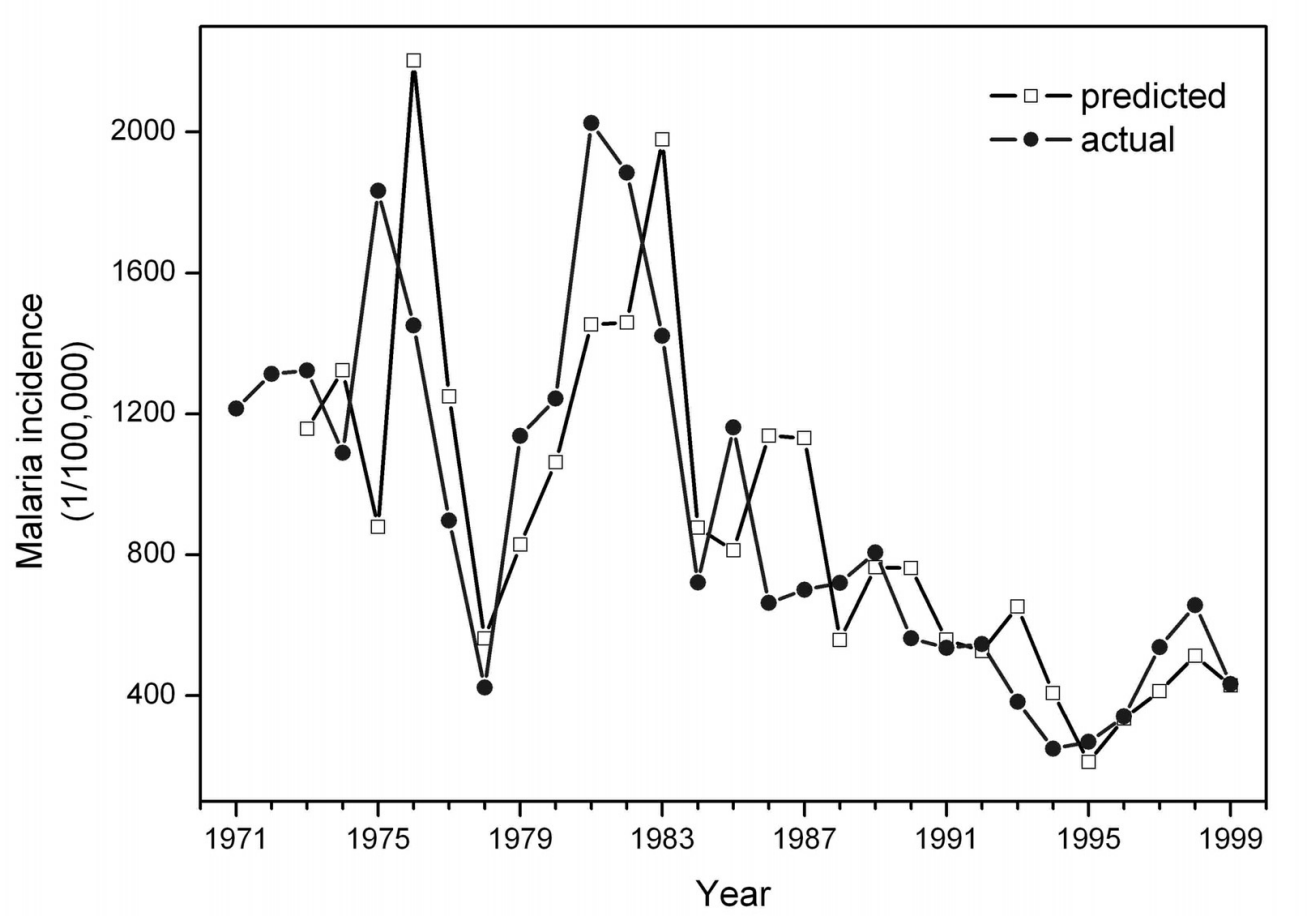
Regressive forecasts of annual malaria incidence in Mengla, 1971–1999.

At the time scale of years, the 29-year series (Figure 4) were used to identify an ARIMA *(1,1,0) *model for the annual malaria incidence (Table 2). The only weather variable associated with annual malaria incidence is the fog day frequency with a lead time of one year (*β *= 0.003, *p *= 0.008). The regressive forecast chart indicates that the predicted value and the actual incidence of annual malaria incidence matched reasonably well. The incidence of malaria from 1998 to 1999 was theoretically predicted by the model and validated by the actual values (Figure 5).

**Table 2:** ARIMA regression of the logarithmic annual malaria incidence (1971–1999) on the fog day frequency in Mengla, China

Variable	*β*	S.E.	*p*
Auto-regression	-0.564	0.176	0.004
Fog day frequency (lag 1)	0.003	0.001	0.008

## Discussion

Seasonal dependence (seasonality) is apparent in the time series of malaria incidence in this tropical rain forest study area. Besides the major incidence peak in the rainy season, there is also a minor peak in the dry-cool season. A similar bimodal annual pattern of malaria is seen in the Limbe River valley of northern Haiti, with two periods of high incidence in June and July in summer and December and January in winter [22]. Seasonality is one type of autocorrelation, which refers to the correlation of a time series with its own past and future values. Cross-correlations are correlations between two time series shifted in time relative to one another. In the presence of autocorrelation in the individual series, the estimated cross-correlation function may be distorted [23]. Prior to the examination of interdependency between the climate and malaria time series, seasonal autocorrelation has to be removed by prewhitening. Successful modeling using a seasonal ARIMA model in this study was the basis for the examination of the effects of climate variability on malaria transmission.

This study fails to find an association between relative humidity and malaria incidence in the tropical rain forest area of Mengla County, China. Provided that relative humidity has a linear dose-response effect on fluctuations in malaria incidence, this effect would emerge also in fluctuations in the monthly data after filtering out the seasonal component. A high relative humidity lengthens the life of the mosquito and helps the parasite to complete the necessary life cycle so that it can transmit the infection. When the relative humidity drops below 60%, it is believed that malaria transmission cannot occur because of the reduced lifespan of mosquitoes [24]. The relative humidity throughout the year ranges from 69% to 93% (Figure 2), so the relative humidity is probably not a limiting factor for malaria transmission in this tropical rain forest area.

Although rainfall generally increases the number of breeding places for mosquitoes, the current study failed to show rainfall as a precipitating factor for malaria transmission. This is consistent with a few studies in the literature [10,25-27], which find a negative or neutral effect of rainfall. Provided that rainfall has a linear dose-response effect on fluctuations in malaria incidence, this effect would emerge after filtering out the seasonal component. The inconsistent relationship between rainfall and malaria incidence could result from the saturating effect of rainfall, for an increase in rainfall fails to produce additional malaria cases when aquatic breeding sites are not limiting for mosquitoes [28]. In addition, heavy rainfall or storms may destroy existing breeding places, interrupt the development of mosquito eggs or larvae, or simply flush the eggs or larvae out of the pools.

Both minimum temperature and maximum temperature were positively associated with malaria incidence in the study area. This finding agrees with the finding of other field studies [6,7,12,14,28-30] in which temperature is reported to be a precipitating factor for malaria transmission. This is biologically plausible because temperature affects three aspects of malaria transmission: 1) the survival and reproduction rates of *Anopheles*; 2) the intensity, particularly the biting rate, of *Anopheles *activity; and 3) the development, survival, and reproduction rates of the *Plasmodium *within *Anopheles*.

The lead time between minimum temperature and malaria incidence is one to two months in this area. This is similar to the estimate of a seven- to ten-week lead time for the cold regions and nine- to ten-week lead time for the warmer regions of Ethiopia [28]. In another county of southern China, minimum temperature affects malaria incidence with a one-month lagged effect [12]. The detection of a positive effect of maximum temperature at a long lag of four months is not explainable, to our knowledge, on biological grounds.

The association between minimum temperature and malaria incidence is not constant throughout the year. It appears stronger in the cool months than in the hot months. The one-month delayed effect of minimum temperature on malaria is particularly strong in December, and the two-month delayed effect of minimum temperature on malaria is particularly strong in February. It is likely that temperature is a more important limiting factor for malaria transmission in the cool months than in the hot months. This temporal differentiation of the temperature effect is analogous to reports that temperature increase has a greater effect on malaria transmission in cool areas than in warm areas [28,29].

The fog day frequency in one month is associated with malaria incidence with a seven-month delayed effect. The overall association is driven particularly by the cross-correlation between fog frequency in October and malaria incidence in May of the following year. This is the first time that the effect of fog on malaria transmission has been reported in the literature. The possibility cannot be ruled out that this is a spurious finding since multiple lags (one to 12 months) were tested, but this association is biologically plausible. Moreover, the delayed effect of fog frequency on malaria was also revealed at the time scale of years.

Fog precipitation is an important water input in many mountainous and coastal environments. The positive effect of fog on malaria transmission may involve providing water input and maintaining aquatic breeding sites for mosquitoes in vulnerable times when there is little rainfall in the six-month dry season (November-April). The study area is located in a tropical seasonal rain forest region in south-west China, where the daily fog drip amount is 0.38 mm on average [31]. Radiation fog forms during the night when cooling caused by long-wave radiation lowers the air temperature to or below the dew point. In the dry-cool and dry-hot seasons of this rain forest, fog drip represents up to 49% and 33%, respectively, of the total precipitation (rainfall and fog) [31]. The dry-cool season and the dry-hot season have average daily fog duration of 11 and 9 h, respectively, while the rainy season has 6 h [32].

The lead time from fog events and malaria is seven months at the time scale of months, and one-year at the time scale of years. The delayed effect is mainly driven by the association between fog events in October and malaria incidence in May of the following year. The lack of an immediate effect of the fog events may be due to the negative correlation of fog frequency and concurrent minimum temperatures. Radiation fog forms only when the air temperature reaches or falls below the dew point. Fog precipitation may contribute to maintaining aquatic breeding sites for mosquitoes in cool months, but its effect on malaria transmission emerges only when the temperature and humidity are also optimum for malaria transmission. The delayed effect of fog events on malaria transmission may also involve the interaction of other hydrology and ecology factors in this tropical seasonal rain forest area.

The current study has two limitations. First, *P. vivax *and *P. falciparum *malaria cases were pooled together when malaria incidence was calculated. Detailed data on each *Plasmodium *were not available for the study period. The ratio of *P. vivax *malaria cases to *P. falciparum *malaria cases was roughly 4:1. Second, potential confounding factors, such as land cover change and public health intervention measures, may have influenced malaria incidence and may have been associated with the weather factors examined in this study. Because of the lack of historical data, these factors were not adjusted for in the regression modeling.

## Conclusion

Minimum temperature, maximum temperature, and fog day frequency are predictors for malaria incidence in the tropical seasonal rain forest area of Mengla County, China. The effect of minimum temperature on malaria incidence is greater in the cool months than in the hot months. The fog day frequency in October has a positive effect on malaria incidence in May of the following year. On the time scale of years, fog day frequency has a one-year delayed effect on the annual incidence of malaria. These findings should be considered in the prediction of future patterns of malaria for similar tropical rain forest areas worldwide.

## Authors' contributions

LWT and YB conceived the study, undertook statistical analysis and drafted the manuscript, SCH, EYYC and JJYS made major contributions to the study design and statistical analysis, WJL and SSZ initiated the study and made weather data and malaria incidence data available, SL and WBG participated in the statistical analysis and helped to draft the manuscript. All authors contributed to the writing of the manuscript and approved the submitted version of the manuscript.
